# Healthcare resource utilization and costs after initiating direct-acting oral anticoagulants or low molecular weight heparins in patients with venous thromboembolism

**DOI:** 10.1177/1358863X241305097

**Published:** 2025-01-06

**Authors:** Godwin Okoye, Kenechukwu C Ben-Umeh, Anton LV Avanceña, Eberechukwu Onukwugha

**Affiliations:** 1 Health Outcomes Division, College of Pharmacy, The University of Texas at Austin, Austin, TX, USA; 2 Department of Pharmacotherapy, College of Pharmacy, University of Utah, Salt Lake City, UT, USA; 3 Department of Internal Medicine, Dell Medical School, The University of Texas at Austin, Austin, TX, USA; 4 Department of Practice, Sciences, and Health Outcomes Research, School of Pharmacy, University of Maryland, Baltimore, MD, USA

**Keywords:** Direct oral anticoagulant (DOAC), healthcare cost, healthcare use, low molecular weight heparin, venous thromboembolism (VTE)

## Abstract

**Background:**

Venous thromboembolism (VTE) can lead to significant healthcare resource utilization (HcRU) and costs. First-line treatments such as direct-acting oral anticoagulants (DOAC) and low molecular weight heparin (LMWH) are utilized for VTE management. There are limited observational studies to determine which first-line drug for VTE is associated with lower HcRU and cost. Therefore, we sought to compare HcRU and costs of commercially insured patients with VTE who initiated DOAC or LMWH in the US.

**Methods:**

We utilized Merative MarketScan Research Database (2016–2021) to identify adults initiating DOAC or LMWH for VTE. Baseline measures were assessed 12 months prior to the index date of drug initiation. Inverse probability of treatment weighting was used to control confounding. For HcRU, logistic regression was used to model emergency room and inpatient visits and the negative binomial count model was used for outpatient visits. The average marginal effect for total healthcare cost comparing DOAC with LMWH users was estimated using a generalized linear model. HcRU and costs were evaluated for 12 months posttreatment initiation.

**Results:**

DOAC users had lower odds of inpatient visits (adjusted odds ratio [aOR] 0.53, 95% CI 0.46 to 0.59), emergency room visits (aOR 0.86, 95% CI 0.73 to 0.99), and outpatient visits (adjusted incident rate ratio 0.52, 95% CI 0.50 to 0.54) in comparison to LMWH users. DOAC users had lower total healthcare costs of −$9573 (95% CI −$11,149 to −$7997) (US dollars).

**Conclusion:**

This cohort study suggests that DOAC use is associated with fewer inpatient, outpatient, and emergency room visits, and lower healthcare costs compared to LMWH use for VTE management.

## Background

Venous thromboembolism (VTE), which includes deep vein thrombosis and pulmonary embolism, are common blood clots that occur after surgery, injuries, and as a consequence of chronic conditions like stroke, cancer, and bleeding disorders.^1,2^ VTE is a leading cause of morbidity and mortality; a significant proportion of hospital-related disability-adjusted life years lost are attributable to VTE.^3^ VTE has a 30% chance of recurrence within 10 years of onset.^3,4^ In the US, VTE has an annual incidence of about 1 case per 1000 adults, causes over 500,000 hospitalizations, and is a leading cause of preventable hospital deaths.^3–6^ In addition to its morbidity and mortality, VTE leads to substantial clinical and economic burden for the patients and the healthcare system.^7–9^ Patients with VTE often visit outpatient clinics for diagnosis and treatment.^8^ Severe cases may require emergency room visits, which can lead to hospitalization.^8,9^ For patients with unprovoked recurrent VTE, lifelong treatment is recommended.^9^

VTE is primarily treated with anticoagulants.^10^ Anticoagulants like warfarin, unfractionated heparins, and low molecular weight heparins (LMWHs) were the primary treatments for VTE.^10,11^ Recent guidelines, however, have recommend the use of direct-acting oral anticoagulants (DOACs) as first-line treatment for VTE in most patients.^12,13^ DOACs have demonstrated higher efficacy in the management of VTE and are also commonly prescribed for VTE because of the convenience of an oral route of administration.^14,15^ However, DOAC use is not without risks of adverse events like stroke and bleeding events that may increase healthcare utilization and cost.^16,17^

Some studies have compared the risk and benefits of initiating DOAC and LMWH for other indications, yet there has been limited evidence regarding the economic benefits of using either of these first-line drugs for VTE.^15–17^ These economic benefits include a reduction in outpatient, inpatient, and emergency room visits due to adverse events related to anticoagulant initiation. The SELECT-D randomized controlled trial (RCT) has shown that rivaroxaban (DOAC) is associated with lower recurrence of VTE compared to dalteparin (LMWH).^18^ Another RCT comparing edoxaban (DOAC) with dalteparin (LMWH) corroborated this result.^19^ However, we do not know if these results from RCTs translate to lower healthcare resource utilization (HcRU) and cost in the real-world setting.

In this cohort study, we utilized real-world data to compare HcRU and costs between patients who received DOAC and LMWH as first-line VTE treatment. Based on RCT data that showed lower recurrence of VTE among DOAC users, we hypothesized that DOAC will be associated with reduced use of healthcare services compared to LMWH. Findings from this study can inform prescribers’ clinical decision making and future guidelines and policies by payers and professional societies related to VTE management.

## Methods

### Data source and study design

This retrospective cohort study was conducted using the Merative MarketScan Commercial Claims and Encounters Database from January 1, 2016 to December 31, 2021. Each year the MarketScan database contains information on over 25 million individuals with an employer-sponsored medical and/or pharmacy plan. The data include employers, payers, and providers from all 50 states and the District of Columbia, and enrollees are generally representative of the commercially insured population in the US. MarketScan includes deidentified data on patient demographics, type of health plan, length of enrollment, diagnoses, and medical claims for inpatient, outpatient, and pharmacy services as well as their associated costs. This study does not involve human subjects and was deemed exempt by the Institutional Review Board of the University of Texas at Austin (STUDY00005236).

### Study population and exposure

We included commercially insured adults aged 18–64 years at the time of a VTE-related healthcare encounter. VTE was defined based on a primary or secondary diagnosis of VTE in at least one inpatient or two outpatient claims. VTE was identified using the following *International Classification of Diseases, 10*^th^ *Revision, Clinical Modification* (ICD-10-CM) codes: 182.xx, I26.xx, I80.xx, O87.xx, O88.xx, and O22.xx. These ICD-10-CM codes have been validated by Molander et al. with a positive predictive value of 87% (95% CI 80% to 98%) for identification of VTE.^20^

Patients included in this study were required to have initiated DOAC or LMWH within 90 days of the first qualifying claim for VTE. We defined the index date as the date of DOAC or LMWH initiation. DOAC users were individuals who initiated rivaroxaban, apixaban, edoxaban, and dabigatran; LMWH users were individuals who initiated enoxaparin and dalteparin. We identified DOAC and LMWH use based on national drug codes (NDCs) for these drugs in the outpatient drug claims file. We excluded individuals who had any other anticoagulant use prior to the index date of DOAC or LMWH initiation. We also excluded individuals with concurrent claims for DOACs and LMWHs during the month of the index date. We limited our sample to patients who had 12 months of continuous enrollment prior to and after their index date. Patients with any claims for VTE or any anticoagulant use were excluded 12 months prior to DOAC or LMWH initiation. Thus, the earliest index date was January 1, 2017, and the last index date was December 31, 2020 (Supplemental Appendices 1 and 2).

### Baseline covariates

Baseline characteristics like age, sex, geographic region, and insurance type was measured on the index date. We also compared comorbidities associated with VTE during the 12-month baseline period between DOAC and LMWH users. These comorbidities included atrial fibrillation, cardiovascular diseases, chronic obstructive pulmonary disease (COPD), COVID-19, diabetes, hyperlipidemia, hypertension, liver disease, major bleeding, malignant cancer, obesity, osteoporosis, renal disease, stroke, and thrombocytopenia (Supplemental Appendix 3). We also assessed the comorbidity burden using the Charlson Comorbidity Index (CCI) (Supplemental Appendix 4).^21^ We categorized individuals into one of four CCI categories based on the individual’s CCI score: 0 = none, 1 = one comorbidity, 2 = two comorbidities, and 3+ = three or more comorbidities. Time to the initiation of anticoagulants was also recorded.

### Confounder adjustment

We utilized propensity score methods to control measurable sources of confounding.^22^ The propensity score in this study was estimated using logistic regression and is defined as the conditional probability of receiving DOACs given a set of baseline covariates. The estimated propensity scores were utilized to generate an inverse probability of treatment weights (IPTW).^23^ IPTW was used to balance the distribution of observed confounding variables across the comparison groups, making DOAC users comparable to LMWH users. We stabilized the IPTW population using the marginal prevalence of treatment received.^24^ All baseline characteristics were included in the propensity score model except geographical region. We also included the type of VTE into the model (i.e., pulmonary embolism/deep vein thrombosis) and the time to treatment initiation of the anticoagulants being studied. We also included separate indicator variables to identify the comorbidities utilized for the CCI. These include myocardial infarction, congestive heart failure, peripheral vascular disease, cerebrovascular disease, dementia, COPD, rheumatic disease, peptic ulcer, mild liver disease, diabetes without complications, diabetes with complications, paraplegia and hemiplegia, renal disease, cancer, moderate or severe liver disease, metastatic carcinoma, and HIV/AIDS.

The performance of the confounder adjustment was evaluated by assessing the distribution of propensity scores and stabilized weights via density plots (Supplemental Appendix 5). We utilized standardized mean differences (SMD) in patient characteristics to ascertain covariate balance between DOAC and LMWH users. A 0.1 value for SMD was utilized as a cut-off to establish covariate balance.^25^

### Study outcomes

The outcomes for this study were VTE-related HcRU and cost. We conducted the study from a third-party payer perspective. The components of HcRU included inpatient, outpatient, and emergency room visits associated with a VTE claim. The cost component included the total dollar amount paid by a health plan to a provider or facility for healthcare services delivered. Cost was adjusted to 2022 US dollars using the medical component of the Consumer Price Index.^26^ HcRU and costs were assessed up to 12 months after the index date of initiating DOACs or LMWHs. We utilized the intention-to-treat approach where the first anticoagulant initiated by patients were considered their index medication. Costs were compared between DOAC and LMWH users to estimate the average marginal effect of using DOAC in comparison to LMWH users.

### Sensitivity analysis

We conducted an analysis to determine VTE-related cost of hospitalization for patients in our sample with active cancer. These individuals were identified and analyzed as a clinically relevant subgroup. Our definition of active cancer was (1) having a medical claim for cancer; (2) being hospitalized; and (3) receiving any antineoplastic or radiation therapy.

### Statistical analysis

We utilized descriptive statistics to summarize the patients’ baseline characteristics separately for DOAC and LMWH users. We measured the 12-month mean and median VTE-related inpatient, outpatient, and emergency room visits separately in each group. We also calculated the mean and median overall costs and costs for each type of healthcare use over 12 months. Statistical differences between the two groups were evaluated using Student’s *t*-test for continuous variables, Pearson’s chi-squared test for binary variables, and the Mantel–Haenszel chi-squared test for nominal variables.

Inpatient and emergency room visits were infrequent and best modelled using a logistic regression model which models the likelihood of DOAC users to have a VTE-related inpatient or emergency room visit, respectively, in comparison to LMWH users. For outpatient visits, we employed various count models and selected the negative binomial model which had the best model fit based on the Akaike Information Criterion. This model was utilized to estimate the incident rate ratio of VTE-related outpatient visits during the follow-up period for DOAC users compared to LMWH users.

For healthcare costs, we first presented descriptive statistics stratified by HcRU category and by type of anticoagulant medication. We used a quantile–quantile plot and probability plot to determine the distribution of the cost data.

To compare costs between DOAC and LMWH users, we used a two-part model that accounts for zero values and skewed nonzero values. The first part used logistic regression to estimate the likelihood of incurring any cost (i.e., odds of having a strictly positive cost vs zero cost). The second part used a generalized linear model with a gamma distribution and a log link to model positive total costs among DOAC users compared to LMWH users. The average marginal effect of utilizing DOACs versus LMWHs on the total direct healthcare cost of patients with VTE was estimated by computing the marginal effect for each individual and then averaging over the individual marginal effects.^27^ The 95% CIs for the average marginal cost were calculated using the delta method.

We used the modified Park test to test for misspecification of the regression models (Supplemental Appendix 6). All statistical analysis was conducted using SAS version 9.4 (SAS Institute, Cary, NC, USA), and a *p*-value less than 0.05 was considered statistically significant.

## Results

### Baseline characteristics

We included 20,958 patients with VTE (48% with pulmonary embolism and 52% with deep vein thrombosis); of this sample, 16,884 (80%) were DOAC users and 4074 (20%) were LMWH users (Supplemental Appendix 1). After applying our inclusion criteria, all patients in the DOAC group were only on apixaban. Table 1 summarizes the baseline characteristics of the study participants. DOAC users had a higher mean age than LMWH users (50.72 vs 47.72 years). The median time to initiation of DOACs and LMWHs was 3 and 4 days, respectively. Patients with VTE were more likely to be women (64.11% among DOAC users and 68.85% for LMWH users). Based on the CCI, more DOAC users had zero comorbidities compared to LMWH users (63.22% vs 56.16%). By contrast, more LMWH users had three or more comorbidities than DOAC users (13.7% vs 8.12%). More LMWH users were cancer survivors compared to DOAC users (13.5% vs 4.68%). After applying the IPTW method for confounder adjustment, we established adequate covariate balance across all covariates included in the model (Table 1).

**Table 1.** Unweighted and weighted demographic and clinical characteristics of patients with venous thromboembolism treated with DOACs or LMWHs.

**Characteristics**	Unweighted sample			Weighted sample		
	DOAC *n* = 16,884	LMWH *n* = 4074	SMD^a^	DOAC *n* = 16,908	LMWH *n* = 4066	SMD^a^
Age, years, mean (SD)	50.72 (10.18)	47.72 (11.32)	0.278	50.12 (10.59)	50.12 (10.31)	< 0.001
Time to treatment initiation, days			0.190			0.047
Mean (SD)	15.15 (21.88)	19.72 (31.92)		14.84 (27.69)	16.02 (22.84)	
Median (IQR)	3 (1, 20)	4 (1, 24)		3 (1, 20)	4 (1, 22)	
Sex, *n* (%)			0.101			0.025
Male	6060 (35.89)	1269 (31.15)		5924 (35.04)	1473 (36.23)	
Female	10,824 (64.11)	2805 (68.85)		10,984 (64.96)	2593 (63.77)	
Region, *n* (%)						
Northeast	1144 (6.78)	404 (9.91)	N/A^b^	–	–	N/A^b^
Midwest	2985 (17.68)	891 (21.87)		–	–	
South	6008 (35.58)	1136 (27.88)		–	–	
West	1162 (6.88)	402 (9.87)		–	–	
Unknown	5585 (33.07)	1241 (30.46)				
CCI, *n* (%)						
0	10,674 (63.22)	2288 (56.16)	0.219	10,419 (61.62)	2450 (60.25)	0.046
1	3138 (18.59)	675 (16.57)		3076 (18.20)	761 (18.72)	
2	1701 (10.07)	553 (13.57)		1842 (10.90)	472 (11.61)	
⩾ 3	1371 (8.12)	558 (13.7)		1570 (9.28)	383 (9.42)	
Comorbidities, *n* (%)						
Atrial fibrillation	408 (2.42)	79 (1.94)	0.033	394 (2.33)	98 (2.41)	0.005
Cancer diagnosis	791 (4.68)	550 (13.5)	0.310	1114 (6.59)	274 (6.75)	0.006
Cardiovascular diseases	2100 (12.44)	437 (10.73)	0.053	2051 (12.13)	506 (12.44)	0.009
COPD	335 (1.98)	82 (2.01)	0.002	338 (2.00)	78 (1.93)	0.005
COVID-19	1311 (7.76)	80 (1.96)	0.272	1121 (6.63)	274 (6.73)	0.004
Deep vein thrombosis	8442 (50.00)	2363 (58.00)	0.161	8713 (51.53)	2051 (50.44)	0.022
Diabetes	1587 (9.4)	333 (8.17)	0.043	1548 (9.16)	387 (9.52)	0.012
Hyperlipidemia	2152 (12.75)	353 (8.66)	0.132	2021 (11.95)	504 (12.41)	0.014
Hypertension	4260 (25.23)	867 (21.28)	0.093	4145 (24.52)	1055 (25.94)	0.033
Liver diseases	411 (2.43)	113 (2.77)	0.029	423 (2.50)	101 (2.48)	0.001
Major bleeding	1291 (7.65)	424 (10.41)	0.096	1401 (8.29)	366 (8.99)	0.025
Obesity	2601 (15.41)	631 (15.49)	0.002	2616 (15.47)	660 (16.24)	0.021
Osteoporosis	332 (1.97)	79 (1.94)	0.002	330 (1.95)	78 (1.91)	0.003
Pulmonary embolism	8442 (50.00)	1711 (42.00)	0.161	8195 (48.47)	2015 (49.56)	0.022
Renal disease	1465 (8.68)	348 (8.54)	0.015	1469 (8.69)	371 (9.13)	0.015
Stroke	233 (1.38)	63 (1.55)	0.014	239 (1.41)	60 (1.47)	0.005
Thrombocytopenia	1650 (9.77)	508 (12.47)	0.086	1760 (10.41)	436 (10.73)	0.010

Our stabilized IPTW model slightly up-weighted the DOAC group and down-weighted the LMWH group.

a We used a cut-off of < 0.1 to establish adequate covariate balance between DOACs and LMWH.

b Covariate not added into the Propensity Score model.

CCI, Charlson Comorbidity Index; COPD, chronic obstructive pulmonary disease; DOAC, direct-acting oral anticoagulant; IPTW, inverse probability of treatment weights; LMWH, low molecular weight heparin; N/A, not applicable; SMD, standard mean difference.

### Descriptive analysis of healthcare resource utilization (HcRU) and costs

Table 2 describes VTE-related HcRU. DOAC users had fewer inpatient visits than LMWH users within a 12-month follow up (0.08 vs 0.23, *p* < 0.001). An emergency room visit was a rare outcome in our data set. DOAC users compared to LMWH users had an average of 0.06 versus 0.08 visits (*p* = 0.051). DOAC users had fewer outpatient visits within 1 year (3.77 vs 6.91, *p <* 0.001).

**Table 2.** 12-month venous thromboembolism-related HcRU in patients treated with DOACs or LMWHs.

	DOAC *n* = 16,884			LMWH *n* = 4074			
	Mean (SE)	Median (IQR)	Range	Mean (SE)	Median (IQR)	Range	*p*-value^a^
Inpatient visits	0.08 (0.003)	0 (0, 0)	0–6	0.23 (0.01)	0 (0, 0)	0–12	< 0.001
Emergency room visits	0.06 (0.003)	0 (0, 0)	0–6	0.08 (0.01)	0 (0, 0)	0–3	0.051
Outpatient visits	3.77 (0.04)	3 (1, 5)	0–55	6.91 (0.13)	3 (1, 9)	0–52	< 0.001

a *p*-value comparing mean values from both groups.

DOAC, direct-acting oral anticoagulant; HcRU, healthcare resource utilization; LMWH, low molecular weight heparin.

Table 3 describes VTE-related healthcare costs. DOAC users had a mean (SE) cost of $7600 ($299) and LMWH users had a mean (SE) cost of $16,767 ($1039). A breakdown of costs by HcRU shows that inpatient cost constituted a larger proportion of total costs among LMWH users than DOAC users (72.23% vs 54.22%). Mean inpatient costs were also higher among LMWH users than DOAC users ($12,111 vs $4121). Emergency room visits constituted 1.24% of total costs for DOAC users compared to 0.35% for LMWH users. The mean (SE) emergency room-related cost was $94 ($18) for DOAC users and $59 ($9) for LMWH users. Outpatient visits constituted 44.54% of total costs in DOAC users compared to 27.42% in LMWH users. The mean (SE) outpatient cost for DOAC users was $3385 ($164), whereas LMWH users had a mean (SE) cost of $4597 ($245).

**Table 3.** 12-month venous thromboembolism-related healthcare costs in patients treated with DOACs or LMWHs.

	DOAC *n* = 16,884				LMWH *n* = 4074			
	Mean (SE)	Median (IQR)	Range	%	Mean (SE)	Median (IQR)	Range	%
Total cost	7600 (299)	579 (47, 3119)	0–1,034,880	100	16,767 (1039)	772 (17, 8236)	0–1,071,152	100
Cost per HcRU category								
Inpatient visits	4121 (244)	0 (0, 0)	0–998,280	54.22	12,111 (971)	0 (0, 0)	0–1,071,152	72.23
Emergency visits	94 (18)	0 (0, 0)	0–205,183	1.24	59 (9)	0 (0, 0)	0–12,697	0.35
Outpatient visits	3385 (164)	471 (165, 2261)	0–1,034,880	44.54	4597 (245)	3086 (420, 3086)	0–627,629	27.42

All costs are in 2022 US dollars ($).

DOAC, direct-acting oral anticoagulant; HcRU, healthcare resource utilization; LMWH, low molecular weight heparin.

### Regression analysis of HcRU

The crude and adjusted results of the HcRU regression analyses are shown in Table 4. DOAC users were less likely to have inpatient visits, with an adjusted odds ratio (aOR) of 0.53 (95% CI 0.46 to 0.59, *p* < 0.001) compared to LMWH users. DOAC users also had lower odds of emergency room visits compared to LMWH users (aOR 0.86, 95% CI 0.73 to 0.99, *p* = 0.047). For outpatient visits, DOAC users had a lower incidence rate than LMWH users, with an adjusted incident rate ratio of 0.52 (95% CI 0.50 to 0.54, *p* < 0.001).

**Table 4.** Regression analysis of HcRU data.

**Logistic regression**	Crude			Adjusted		
	Odds ratio	95% CI	*p*-value	Odds ratio	95% CI	*p*-value
Inpatient visits	0.39	0.35–0.45	< 0.001	0.53	0.46–0.59	< 0.001
Emergency room visits	0.83	0.71–0.97	0.026	0.86	0.73–0.99	0.047
**Negative binomial count model**	Incident rate ratio	95% CI	*p*-value	Incident rate ratio	95% CI	*p*-value
Outpatient visits	0.57	0.54–0.59	< 0.01	0.52	0.50–0.54	< 0.001

Adjusted results were obtained from the weighted population.

DOAC, direct-acting oral anticoagulant; HcRU, healthcare resource utilization; LMWH, low molecular weight heparin.

### Regression analysis of healthcare costs

We utilized the two-part model to make inference on the odds of observing a nonzero cost and the average marginal effect of DOAC use in comparison to LMWH use. These results are presented in Table 5. The logistic regression shows that nonzero costs were more likely to be observed with an OR 1.18 (95% CI 1.08 to 1.29, *p* < 0.001).

**Table 5.** Regression analysis of healthcare cost data.

Two-part model		95% CI	*p*-value
**Logit model**			
Nonzero cost, OR	1.18	1.08 to 1.29	< 0.001
**GLM (total HcRU cost)**			
Treatment estimate			
DOAC	11,976	11,554 to 12,397	
LMWH	21,549	20,030 to 23,068	
**Average marginal effect** ^a^			
DOAC vs LMWH	–9,573	–11,149 to −7,997	< 0.001

All costs are in 2022 US dollars ($).

a 95% CI was calculated using the delta method.

DOAC, direct-acting oral anticoagulant; GLM, generalized linear model; HcRU, healthcare resource utilization; LMWH, low molecular weight heparin.

The generalized linear model with a gamma distribution and a log link were fit to model the nonzero cost. The estimate for the total healthcare cost for DOAC users was $11,976 (95% CI 11,554 to 12,397, *p* < 0.001). For LMWH users, the estimate for the total healthcare cost was $21,549 (95% CI 20,030 to 23,068, *p* < 0.001). The average marginal effect, which is the average difference in total healthcare costs for DOAC users compared to LMWH users, was –$9573 (95% CI −11,149 to −7997, *p* < 0.001). The negative values indicate that DOAC use is associated with lower costs.

### Sensitivity analysis

We found 177 (4.36% of all LMWH users) patients with active cancer who received LMWHs and 232 (1.37% of all DOAC users) patients who received DOACs. With this subsample of patients, we found the overall average cost of VTE-related hospitalization was higher compared to our entire sample. On average, the cost of hospitalization for DOAC users was $22,056 lower in comparison to patients on LMWHs with active cancer (Supplemental Appendix 7).

## Discussion

This retrospective cohort study used healthcare claims data to compare VTE-related HcRU and costs between those who received DOACs and LMWHs as their first-line treatment for VTE. We found that DOAC users were less likely to have VTE-related inpatient and emergency room visits. One of the major drivers of hospitalization is having active cancer, which is on average more expensive to treat than most chronic conditions. DOAC use was also associated with a lower incidence rate of outpatient visits. The lower utilization among DOAC users also translated to significantly lower VTE-related healthcare costs compared to LMWH users (about $9573 less). These findings may provide valuable insights for providers who treat VTE. It may also help payers understand the economic consequences of first-line VTE treatments.

Over time, DOACs have become highly prescribed for VTE due to reasons such as convenience of oral route of administration, minimal drug interactions, shorter half-life, and lack of the necessity for therapeutic drug monitoring.^14,15^ This is evident in our study with a 4:1 ratio of DOACs in comparison to LMWHs. It is imperative to compare DOACs and LMWHs for VTE because VTE and its related complications cause a significant economic burden on patients and the healthcare system. For example, Ruppert et al.^28^ and Grosse et al.^29^ have conducted systematic reviews that showed the significant cost of managing VTE. Ruppert et al. found that an initial VTE event can cost an average of $33,000 per year, and Grosse et al. found that the first-year costs of a VTE event could cost $42,100 per patient. The costs we estimated in this study are lower than prior estimates, which may reflect improvements in the management of VTE due to the use of first-line anticoagulants.

Our study adds to the small body of literature that has compared HcRU and cost among patients with VTE treated with anticoagulant medications. A study conducted by Streiff et al. used claims data from 2013 to 2015 to compare HcRU and costs for patients with cancer-associated thrombosis who received rivaroxaban (DOAC), LMWHs, and warfarin.^30^ Similar to our study, rivaroxaban was associated with fewer inpatient, outpatient, and emergency room visits. DOAC use was also associated with a $12,000 reduction in cost compared to LMWHs. These findings aligned with a recent model-based economic evaluation that found DOACs were more effective and cost-effective than LMWHs.^31^ Another study based on the EINSTEIN trial by Bookhart et al.^32^ investigated hospitalizations after rivaroxaban compared to a composite measure of enoxaparin (LMWH) and warfarin use. They found that rivaroxaban had a 1.6 day mean reduction in length of stay and was $3419 lower in costs. A meta-analysis of RCTs for VTE prophylaxis among DOAC users in comparison to LMWH users for hospitalized patients showed that apixaban (DOAC) had lower odds of clinically relevant bleeding (OR 0.86, 95% CI 0.58 to 1.26) and a cost saving of $39 per patient per day. This meta-analysis demonstrated the noninferiority of DOACs in comparison to LMWHs and recommended additional research with larger cohorts to confirm their findings.^33^ Another meta-analysis by Tao et al. showed that DOAC users had a lower risk of VTE recurrence (hazard ratio [HR] 0.62, 95% CI 0.43 to 0.91). A reduction in major bleeding events and VTE recurrence could potentially lower healthcare use and cost.^34^ Our study utilized real-world data to evaluate healthcare use and cost for patients with VTE, specifically for those treated with DOACs and LMWHs. Our study confirmed that DOAC use is associated with lower HcRU and cost.

Hospitalization is a major driver of HcRU and cost in VTE. In our study, we observed that the proportion of healthcare costs due to VTE-related inpatient visits was 54.22% versus 72.23%, respectively, for DOAC and LMWH users. This finding may be due to a reduction in the risk of recurrent VTE, major bleeding events, and stroke among DOAC users, which are the main drivers of VTE-related hospitalizations.^33,34^ The ADAM-VTE trial demonstrated decreased odds of recurrent VTE for DOACs (OR 0.11, 95% CI 0.01 to 0.78) compared to LMWHs.^35^ The same observation has been reported in other trials, although the effect was not statistically significant.^36–39^ For major bleeding events, most studies suggest conflicting results for DOACs versus LMWHs.^35–38^ A network meta-analysis conducted by Rossel et al. favored DOACs as being less likely to cause recurrent VTE, with a HR of 0.63 (95% CI 0.42 to 0.96), which may translate to a lower risk of VTE-related hospitalization compared to LMWHs.^38^ The findings of our cohort study support the positive association between healthcare use and costs among patients with VTE and a reduction of this cost by initiating treatment.

To our knowledge, this study is the first real-world comparison of HcRU and cost among patients with VTE who receive DOACs and LMWHs. The results of this study are in line with both RCTs for VTE and observational studies for other disease conditions like cancer-associated thrombosis, diabetes-associated thrombosis, and obesity-associated thrombosis.

This study has some limitations. First, it relied on commercial claims data, limiting the generalizability of the findings to the broader US population. Coding errors within claims data may have affected the accuracy of diagnosis variables. Additionally, the study employed an intention-to-treat approach, disregarding switches between medications during the follow-up period, a factor that could impact outcomes. Though common in RCTs for VTE studies, other analytical methods such as per-protocol and as-treated analyses were not considered. Furthermore, the study solely examined healthcare costs, warranting further studies that include VTE-related, nonhealthcare costs like loss of work productivity and caregiving costs. Also, the choice of anticoagulant may vary by the underlying medical conditions of patients, as recommended by various treatment guidelines. Our study did not directly account for this difference.

Our study has many strengths. First, we conducted a real-world study with recent data and more patients than were enrolled in various RCTs that compared DOACs and LMWHs. For instance, the ADAMS VTE trial only had 300 participants, whereas we included over 20,000 real-world users of DOACs and LMWHs for VTE. We utilized IPTW to control confounding factors, enhancing the robustness of our findings. This study also fills the research gap regarding the lack of evidence for DOACs versus LMWHs for patients with VTE specifically. Our findings are timely given the evolving clinical management of VTE and the importance of minimizing its economic burden.

## Conclusion

DOAC use is associated with reduced inpatient, outpatient, and emergency room visits and lower healthcare costs compared to LMWH use in VTE. These findings provide valuable insights for optimizing VTE therapy in clinical practice.

## Supplemental Material

sj-pdf-1-vmj-10.1177_1358863X241305097 – Supplemental material for Healthcare resource utilization and costs after initiating direct-acting oral anticoagulants or low molecular weight heparins in patients with venous thromboembolism Supplemental material, sj-pdf-1-vmj-10.1177_1358863X241305097 for Healthcare resource utilization and costs after initiating direct-acting oral anticoagulants or low molecular weight heparins in patients with venous thromboembolism by Godwin Okoye, Kenechukwu C Ben-Umeh, Anton LV Avanceña and Eberechukwu Onukwugha in Vascular Medicine
