# Supplementary material for: Healthcare resource utilization and costs after initiating direct-acting oral anticoagulants or low molecular weight heparins in patients with venous thromboembolism
Source: Vasc Med. 2025 Jan 6;30(2):197–204. doi: 10.1177/1358863X241305097 (PMC12014951; doi:10.1177/1358863X241305097)
Supplement: sj-pdf-1-vmj-10.1177_1358863X241305097 – Supplemental material for Healthcare resource utilization and costs after initiating direct-acting oral anticoagulants or low molecular weight heparins in patients with venous thromboembolism [file sj-pdf-1-vmj-10.1177_1358863X241305097.pdf]

Supplemental Appendices

Appendix 1: Consort diagram showing results of cohort selection for patients with venous thromboembolism who initiated DOAC or LMWH 2

Appendix 2: Timeline for cohort selection and outcome measurement for patients diagnosed with venous thromboembolism who initiated DOAC or LMWH 3

Appendix 3. Comorbidities assessed during the baseline period 4

Appendix 4. Charlson Comorbidity Index 5

Appendix 5: Propensity score distribution before (A) and after (B) inverse probability of treatment weighting 6

Appendix 6. Modified Park test results 7

Appendix 7: Sensitivity analysis for VTE-related hospitalization for patients with active cancer 8

Appendix 1: Consort diagram showing results of cohort selection for patients with venous thromboembolism who initiated DOAC or LMWH

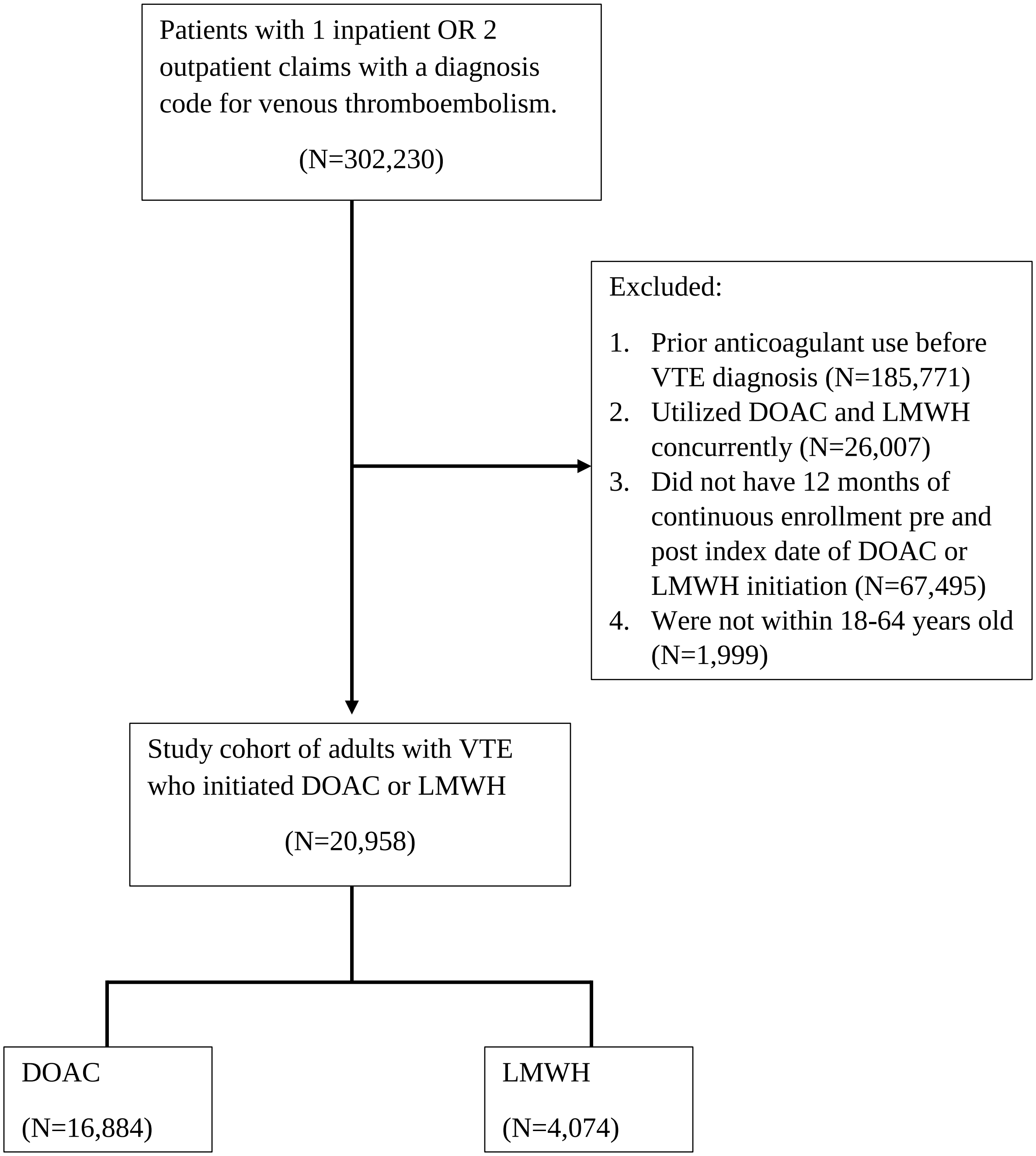

Appendix 2: Timeline for cohort selection and outcome measurement for patients diagnosed with venous thromboembolism who initiated DOAC or LMWH

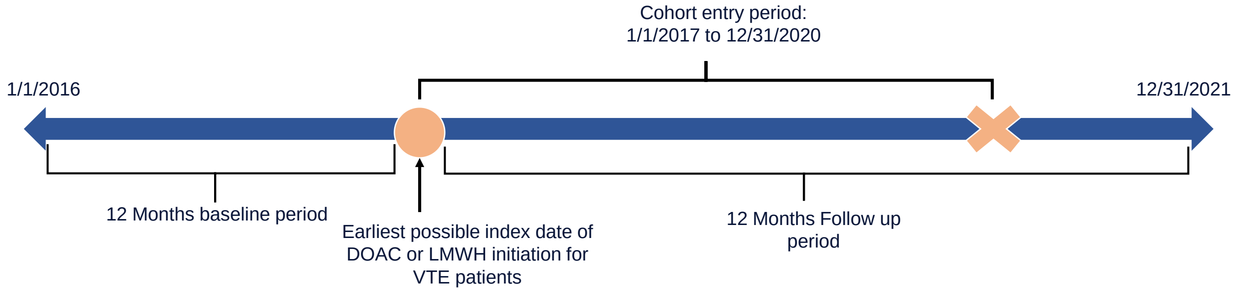

Appendix 3. Comorbidities assessed during the baseline period

S/N	Disease or condition	ICD-10-CM codes
**1**	Stroke	I61.XX- I66.XX, G45.XX
**2**	Major bleeding	I31.XX, I62.XX, K64.XX, I85.XX, R58.XX, K22.XX, K25.XX-K29.XX, S06.XX
**3**	Cardiovascular Disease	I20-122.XX, I24.XX, I25.XX, I50.XX, I70.XX, I71.XX, I73.XX, I77.XX, I79.XX, K55.XX, Z95.XX
**4**	Diabetes	E10-E14.XX
**5**	Hypertension	I10.XX-I16.XX
**6**	Hyperlipidemia	E78.XX
**7**	Obesity	E66.XX
**8**	Chronic Obstructive Pulmonary Disease (COPD)	J41.XX - J44.XX
**9**	Renal disease	N18.XX, I12.0X, I13.1X, E13.XX, N03.2X-N03.7X, N05.7X, N19.XX, N25.0X, Z49.0X, Z91.XX, Z94.0X, Z99.2X, N17.XX-N19.XX, Q60.XX, Q61.XX, R80.XX
**10**	Osteoarthritis	M15.XX, M19.XX, M80.XX, M81.XX, M05.XX, M06.XX
**11**	Thrombocytopenia	D69.XX, D75.XX, D47.XX, D50.XX, D64.XX
**12**	Prior cancer diagnosis	C00.XX–C26.XX, C30.XX–C34.XX, C37.XX–C41.XX, C43.XX, C4A.XX, C44.XX–C58.XX, C60.XX–C96.XX
**13**	Atrial Fibrillation	I48.XX
**14**	COVID-19	U07.1

Appendix 4. Charlson Comorbidity Index

S/N	Disease or condition	ICD-10-CM codes
**1**	Myocardial Infarction	I21.xx, I22.xx, I25.2x
**2**	Congestive Heart Failure	I43.xx, I50.xx, I09.9x, I10.xx, I13.0x, I13.2x, I25.5x, I42.0x, I42.5x, I42.6x, I42.7x, I42.8x, I42.9x, P29.0x
**3**	Peripheral Vascular Disease	I70.xx, I71.xx, I73.1x, I73.8x, I73.9x, I77.1x, I79.0x, K55.9x, Z95.8x, Z95.9x
**4**	Cerebrovascular Disease	G45.xx G46.xx I60-164.xx, I69.xx, H34.0x
**5**	Dementia	F00-F03.xx, G30, F05.1x G31.1x
**6**	Chronic Obstructive Pulmonary Disease (COPD)	J41.XX - J44.XX
**7**	Rheumatic Disease	M05-M06.xx, M32-M34.xx, M31.5x, M35.1x, M35.3x, M36.0x
**8**	Peptic Ulcer Disease	K25-K28.xx
**9**	Mild Liver Disease	B18.xx, K70-K74.xx, K76.xx, Z94.4x
**10**	Diabetes without complications	E10.0x, E10.1x, E10.6x, E10.8x, E10.9x, E11.0x, E12.0x, E12.1x, E12.6x, E12.8x, E12.9x, E13.0x, E14.0x, E14.1x, E14.6x, E14.8x, E14.9x
**11**	Diabetes with complication	E10.2x, E10.3x, E10.4x, E10.5x, E10.7x E11.2x, E12.2x-E12.7x, E13.0x, E14.0x-E14.1x, E14.6x, E14.8x, E14.9x
**12**	Paraplegia and Hemiplagia	G81.xx, G82.xx, G04.1x, G11.4x, G80.1x, G80.2x, G83.4x, G83.9x
**13**	Renal disease	N18.XX, I12.0X, I13.1X, E13.XX, N03.2X-N03.7X, N05.7X, N19.XX, N25.0X, Z49.0X, Z91.XX, Z94.0X, Z99.2X, N17.XX-N19.XX, Q60.XX, Q61.XX, R80.XX
**14**	Moderate or Severe Liver Disease	K70.4x, K71.1x, K72.1x, K72.9x, K76.5x, K76.6x K76.7x, I85.9x, I85.0x, I86.4x, I98.2x
**15**	Metastatic Carcinoma	C77-C80.xx
**16**	Cancer	C00.XX–C26.XX, C30.XX–C34.XX, C37.XX–C41.XX, C43.XX, C4A.XX, C44.XX–C58.XX, C60.XX, C96.XX
**17**	AIDS/HIV	B20-B22.xx, B24.xx

Appendix 5: Propensity score distribution before (A) and after (B) inverse probability of treatment weighting

A.

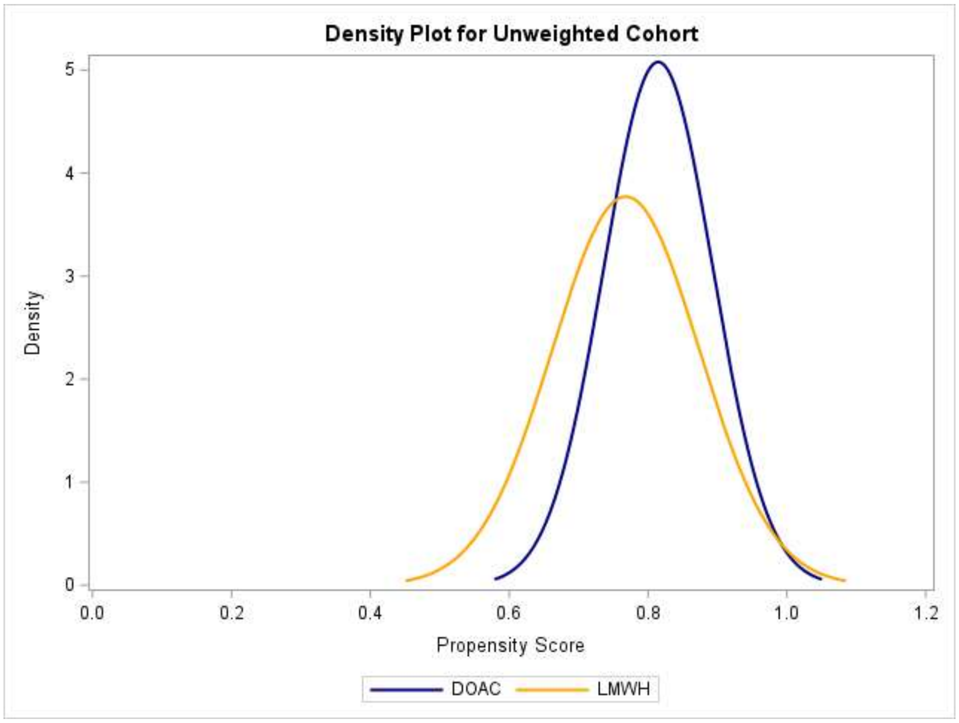

B.

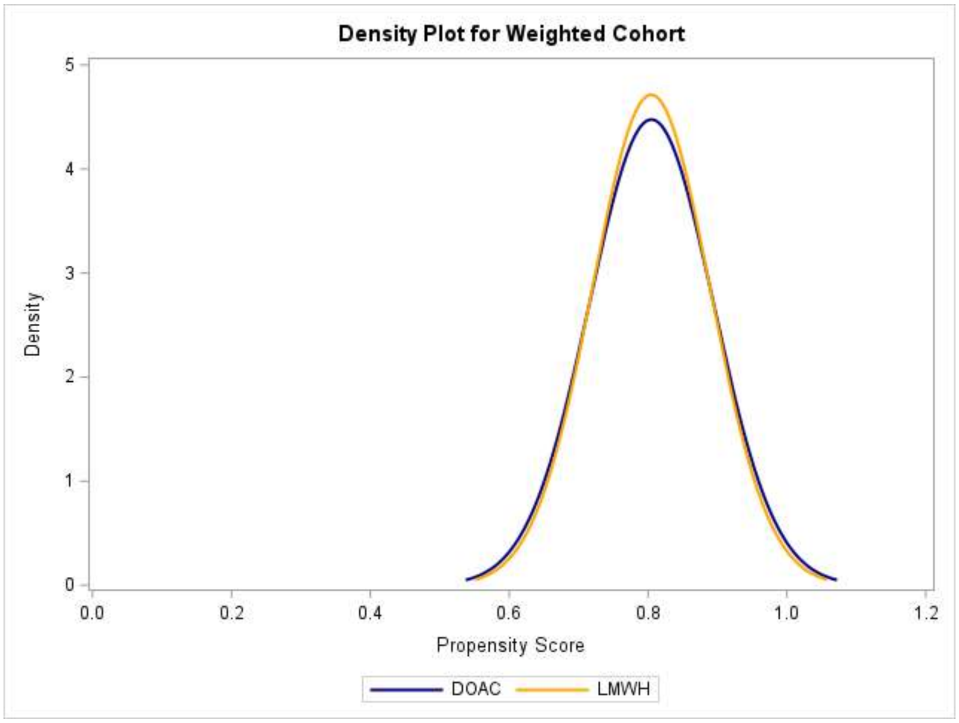

Appendix 6. Modified Park test results

Variable	DF	Parameter estimate	Standard error	t value	Pr > |t|	95% confidence limits	
Intercept	1	-1.15904	0.69986	-1.66	0.0981	-2.53297	0.21488
LnP	1	2.10064	0.06983	30.08	<0.001	1.96355	2.23772

The modified Park test provides constructive guidance on the choice of the distribution for the dependent variable (i.e., the total cost variable). In this case, we utilized the gamma distribution. To conduct this test, we squared the natural logarithm of the residual obtained from regressing our total cost variable on the exposure variable (DOAC vs LMWH). Next, we regress the outcome of the previous step (i.e., the square of the natural logarithm of the residual) on LnP, which is equivalent to regressing the variance on the mean of the predicted values. A value of 2 for the coefficient of LnP indicates data support for choosing the gamma distribution for our total cost variable.

To further show that the cost variable follows an approximate gamma distribution, we presented the figure below showing a graphical assessment of the observed values compared to the predicted values of a gamma and inverse Gaussian distribution.

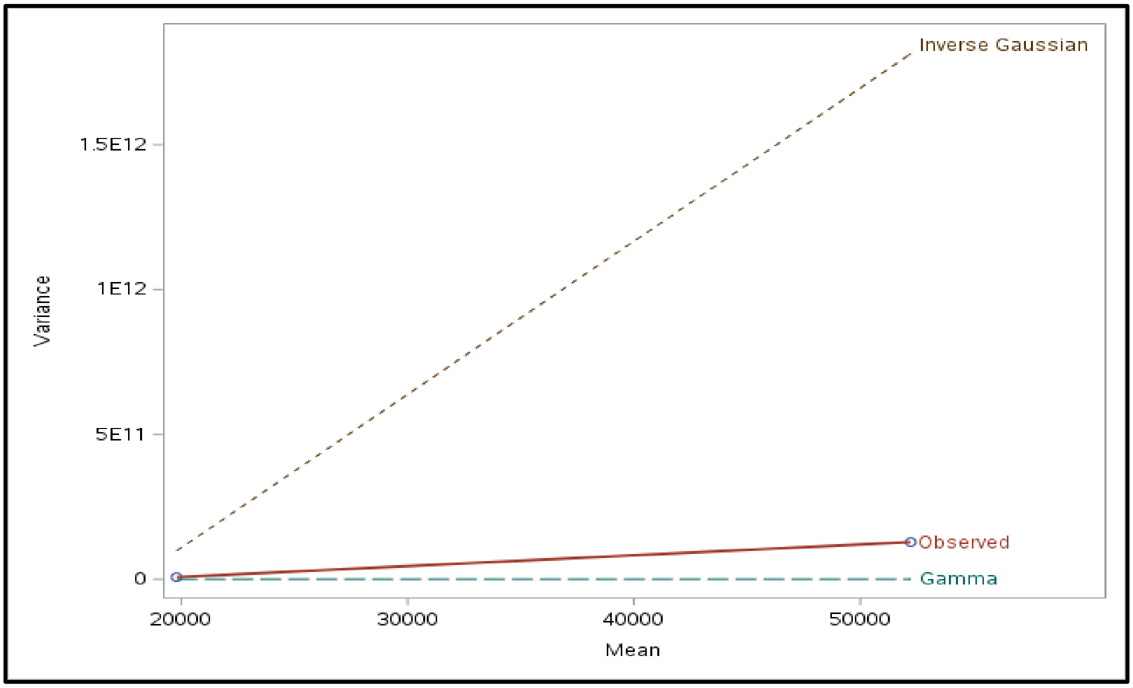

Appendix 7: Sensitivity analysis for VTE-related hospitalization for patients with active cancer

Anticoagulant	Total cost ($)	95% CI ($)	P-value
LMWH	42,580	25,352 – 59,808	0.024
DOAC	20,524	10,054 – 30,994	
